# Supplementary material for: A Comprehensive Evaluation of Salt Tolerance in Tomato (Var. Ailsa Craig): Responses of Physiological and Transcriptional Changes in RBOH’s and ABA Biosynthesis and Signalling Genes
Source: Int J Mol Sci. 2022 Jan 29;23(3):1603. doi: 10.3390/ijms23031603 (PMC8836042; doi:10.3390/ijms23031603)
Supplement: Supplementary file 1 [file ijms-23-01603-s001.zip › ijms-1520603-supplementary.pdf]

Table S1: Effects of salt stress on the gas exchange parameters and the maximum photochemical efficiency (Fv/Fm) of tomato leaves.

Treat	Pn	Gs	Ci	Tr	Fm/Fv
0 day	18.18±1.64a	0.53 ± 0.03a	280.5±8.1a	3.63 ±0.29a	0.72 ±0.01a
3 day	15.87±0.5b	0.25 ± 0.00b	255±10.92b	3.02 ±0.08b	0.64 ±0.05ab
6 day	14.9±0.29b	0.14 ± 0.00c	238.5 ± 5.06b	2.71±0.08b	0.57 ±0.01b
12 day	12.82±1.23c	0.02 ± 0.01d	214.75±17.8c	2.27 ±0.15c	0.32 ±0.09c

Data represent the mean and standard deviation (SD) of three replications. Different letters indicate significant differences according to 'Tukey's HSD test at P ≤ 0.05.

Table S2: List of primers used for qRT-PCR assays

Gene full name	Gene acronym	Accession number	Forward primer (5´-3´)	Reverse primer (5´-3´)
Superoxide dismutase	*SOD*	Solyc02g082590	ACTACTCCCAGTTGCATCCC	CACCAGGAGCAGCCATGATA
Catalase	*CAT*	Solyc12g094620	GCAGCTCCCAGTTAATGCTC	AGCAGGACGACAAGGATCAA
Peroxidase	*POD*	Solyc07g052510	GGTCTGTTCCAATCCGATGC	CACCAGCACTCCCTGTCTTA
Ascorbate peroxidase	*APX*	Solyc06g005150	GGCACTCTGCTGGTACCTAT	GGAGAGAGTGGGAAACTGCT
Glutathione reductase	*GR*	Solyc09g065900	GGAGCCATAGAGGTTGACGA	CTCCTCCCTCCATCAAAGCA
Monodehydroascorbate reductase	*MDHAR*	Solyc08g081530	CGGACAGTTCCGAACAAACA	CCCGTGCAATTCGGTTGTAT
Dehydroascorbate reductase	*DHAR*	Solyc05g054760	GAGGTGAACCCTGAAGGGAA	CCCACAGAGGCAAATTCAGG
Respiratory Burst Oxidase Homolog1	*RBOH1*	Solyc08g081690	TCCAGCACAAGATTACCG	CCTCCATTGCGACGAT
Respiratory Burst Oxidase Homolog-A	*SlRBOHA*	Solyc01g099620	GAGAGTAGGATTCAGCGGT	GCCTCTTTTCGAGCTTGCT
Respiratory Burst Oxidase Homolog-D	*SlRBOHD*	Solyc06g068680	TACACCACCAAATCTAACG	TGCCCAGTGCTTCAATC
Respiratory Burst Oxidase Homolog-E	*SlRBOHE*	Solyc06g075570	GGGTCCTAACATTGTGGTTGT	AGAGTCTCTCCTGCACCTTTA
Respiratory Burst Oxidase Homolog-F	*SlRBOHF*	Solyc07g042460	GCAGCTCAAAACGATCC	CATTGCTCCTCCGACAT
Respiratory Burst Oxidase Homolog-H	*SlRBOHH*	Solyc11g072800	CATGTGTGACAAGAATGGTGATG	TGCTGCATGTTTCTTGAAGTTT
Zeaxanthin epoxidase	*SlZEP*	Solyc02g090890	TCTGGAAGCCCTGTGGAT	TCCGACGCCAAGATAAGC
9-cis-epoxycarotenoid dioxygenase-1	*SlNCED1*	Solyc01g087250	GCTGGATTATGACAAACG	TAAGTGATGTAAGGTGGG
9-cis-epoxycarotenoid dioxygenase-3	*SlNCED3*	Solyc07g056570	CGTGGGCTCTTCGGACTT	ATGGCTTCTGAATCACATCGTA
9-cis-epoxycarotenoid dioxygenase-5	*SlNCED5*	Solyc08g016720	TACTGAAACGGAAAGATTAG	ACTCCACGGGCATAGAAC
Abscisic-aldehyde oxidase3	*SlAAO3*	Solyc01g009230	GGAGGCAAGGCAGTGAAA	CAAGATGTAAGGCGGTGA
Abscisic Acid Insensitive3	*SlABI3*	Solyc06g083590	GGGACTTGTCGTGTTTGG	CACTTTATGTCGGCGTAT

Abscisic Acid Insensitive5	*SlABI5*	Solyc02g088910	GGTGGGAGTAATAATGGTC	TCTGAAGGCTTGATGCTA
Sucrose non-fermenting-1-related protein kinase 2.2	*SlSnRK2.2*	Solyc02g090390	GAAAGAGGCGATAAGATAG	AATAGCGAGATGAGTTGG
Sucrose non-fermenting-1-related protein kinase 2.3	*SlSnRK2.3*	Solyc01g108280	GGCAGTGACAGTAGGACC	TCCATCACAATAGCCAAA
Sucrose non-fermenting-1-related protein kinase 2.6	*SlSnRK2.6*	Solyc01g103940	AGAAATCAAGTCGCACCC	AGACTGAACGGGAAACTG
Pyrabactin Resistance1-like4	*SlPYL4*	Solyc09g015380	CAACCACCACCAGCACCT	ACCCGCACTTCACGGACAG
Pyrabactin Resistance1-like8	*SlPYL8*	Solyc01g095700	TTCGGTTATTACAGTCCA	TCCTCTTTAGTGTTCCCT
binding factor (ABF) members 4	*SlABF4*	Solyc04g078840	ACAGCCACAGCAGCAACC	GCTCTTCCCAAGTCCATC
dehydration-responsive element-2	*SlDREB2*	Solyc09g065990	GGAAGACCAGGAGGGAGA	GGTAGTTTGAAGCCACATA
